# A System of Syphilis

**Published:** 1908-12

**Authors:** 


**A System of Syphilis.** Edited by D'Arcy Power, M.B. Oxon., F.R.C.S., and J. Keogh Murphy, M.D., M.C. Cantab., F.R.C.S. Vol. I. Pp. xxxv., 380. London: Oxford Medical Publications. 1908.

The Oxford Medical Publications have already achieved a well-deserved reputation and a widespread popularity, but the *System of Syphilis* of which this forms the first of six volumes, promises to be the most striking contribution to medical literature.

The names of the joint editors lead us to believe that the work will be of the very highest literary as well as scientific standard, and it is in this belief that we venture to question whether the volume has not been artificially inflated by the use of an exceptionally large type and lavish spacing (*vide* pp. xxv.—xxxii. of the Introduction), while we deplore the appearance of the modern journalistic device of reproducing the signature of each contributor as though he were some herbivorous athlete. Scientific literature stands in no need of these catch-penny methods, which render the unblushing self-advertisement of the *Physician's Legacy* by contrast, inarticulate in its modesty. Apart from these points, which are after all mere questions of taste, the matter in this volume is of the very best.

The Introduction, by Sir Jonathan Hutchinson, gives us the opportunity of studying the mature observations of a true master of clinical method whose contributions to medical and surgical knowledge have covered so vast an area that we, his contemporaries, cannot perhaps give to them the full meed of appreciation that is their due. There is a valuable note of warning, which more than once appears in his introduction: "Unless much caution be exercised . . . it may be reasonably feared that the knowledge of syphilis for practical purposes may be hindered rather than advanced by the recent brilliant discoveries. It might have been safer to continue to base our conclusions on careful observations of what actually occurs in the human subject." With this example of cautiousness, it is to be regretted that Sir Jonathan once again throws the weight of his opinion into the scale of early marriage after acquiring syphilis. It is not an extreme section of faddists who plead for a longer interval than two years between the infection and the permission to marry. His views on beginning mercurial treatment directly the diagnosis is made, and not waiting for the appearance of secondary symptoms, are most acceptable, and in this connection Sir Jonathan points out that the discovery of the spirochæte may place at our disposal a trustworthy means of recognising syphilitic chancres in their earliest stages. Brief mention is made of the controversial question of the relationship of yaws and syphilis, which Sir Jonathan maintains to be the same disease; indeed, one sentence sums up his belief in truly aphoristic fashion: "Whenever an Englishman contracts yaws abroad he comes home with syphilis." As regards treatment, he advocates mercury administered by mouth according to the "small-dose" plan, and expresses his fear lest mercury should suffer a temporary eclipse from less certain and more dangerous therapeutic innovations.

The first four chapters of the volume deal with the history of syphilis, and come from the pen of that able medical historian, Dr. Iwan Bloch. He sums up the evidence of the importation of syphilis into Europe as a new disease in the Middle Ages thus:

"I am of the conviction (which is shared by those who have an exhaustive knowledge of syphilis and its history, such as Unna, Scheube, Fournier, Liebermeister, Binz, and others) that the certain, irrefutable proof of the modern origin of syphilis rests upon nosological and epidemiological grounds; that the facts detailed amply suffice to prove its American origin; that the sudden transformation of the entire medical literature at the end of the fifteenth century furnishes the most complete explanation —but that positive bone discoveries would dispel every lingering and hidden doubt." The care with which every particle of evidence on the subject is sifted and weighed, the impartial summary of contemporary authors, and the judicial reasoning of his final verdict render Dr. Bloch's article one of engrossing interest; though there are occasions when one is tempted to ask whether the voice is not that of a brilliant advocate anxious to compel a decision in his favour rather than that of the open-minded judge. The four plates in this historical section, representing early illustrations of syphilis (Plates I. and II.), the portrait of Fracastorius (Plate III.) and the tailpiece of Fracastorius's poem (Plate IV.), are excellently reproduced and fully justify their inclusion.

"The Microbiology of Syphilis," by Professor Metchnikoff, deals with the many disappointments in pathological research which preceded the results of Schaudinn and Hoffmann, and carry us into the latest phases of our modern knowledge of the part played by the *spirochæta pallida* in the pathogeny of syphilis. Metchnikoff regards the facts as almost conclusive in favour of the ætiological part played by this organism. The description of the natural history of Schaudinn's spirochæte and its connection with trypanosomes and other spirochætes is admirable. So, too, the praise to be bestowed on Chapter X. (Technical Methods of Recognising the Organism) must be the very highest. The value of the ultra-microscope, in demonstating rapidly and simply the unstained organism in a fresh film preparation, is rightly insisted on, and the various more complex staining methods are described in full and intelligible detail. The practical importance of the bacteriology of the disease in the treatment of the syphilitic patient is made very clear. But the translator cannot always be congratulated on having made the author's meaning plain. There is a puzzling sentence at the top of page 69. It is by no means easy to discover to what cells, whether polynuclear leucocytes, or the mononuclear elements described as "embryonic cells," the so-called "plasmatic cells" are intended to be related. Such a blemish as inserting the extra "g" in Noegerath's name on page 88 should not have passed the proof-reader.

Dr. Andrewes contributes an admirable section on the general pathology of syphilis, in which the clinical aspects are never lost sight of. The illustrations are objects of wonder and envy.

Colonel Lambkin's description of the primary lesion and early secondary symptoms in the male falls very far below the remainder of the volume in many respects; personal observations lend weight to a clinical survey, but the personal pronoun is only rarely an adornment. There is a complete absence of any reality in the description of symptoms, never does it compel a mental picture in the imagination of the reader. The section consists mainly of a dogmatic cataloguing of features in a style rendered familiar by only too many students' examination cram-books, and it contrasts poorly with Mr. Shillitoe's illuminating method of dealing with the primary lesion and early secondary symptoms in the female. This is a contribution to clinical medicine which will live even though all the rest of the volume were displaced by the advances of pathology. Congenital syphilis also meets with equally satisfactory treatment at the hands of Dr. Still, whose writings are too well known and highly appreciated to require further commendation.

The illustrations, reproduced from direct colour-photographs, are quite out of the common, and are real aids to the digestion of the letterpress. Finally, the bibliographies clinch the value of the volume, and stimulate the inquiring mind to more extended pasturage.

